# Effects of different proportions of stevia stalk on nutrient utilization and rumen fermentation in ruminal fluid derived from sheep

**DOI:** 10.7717/peerj.14689

**Published:** 2023-01-25

**Authors:** Xia Zhang, Ting Jiao, Shumin Ma, Xin Chen, Zhengwen Wang, Shengguo Zhao, Yue Ren

**Affiliations:** 1 College of Grassland Science, Gansu Agricultural University, Key Laboratory of Grassland Ecosystem, Gansu Agricultural University, Lanzhou, Gansu Province, China; 2 College of Animal Science and Technology, Gansu Agricultural University, Lanzhou, Gansu Province, China; 3 Institute of Animal Husbandry and Veterinary Medicine, Tibet Academy of Agricultural and Animal Husbandry Sciences, Lasa, Tibet Autonomous Region, China

**Keywords:** Stevia stalk, *In vitro*, Sheep, Rumen fermentation, Nutrient digestion

## Abstract

**Background:**

Stevia straw is a byproduct of sugar crop stevia. It is a good feed material because of richness in nutrients and active substances (steviosides and flavonoids). However, due to improper utilization such as piling, burning and so on, it became a large amount of wasted straw resources and lead to environmental pollution.

**Methods:**

We added 0%, 0.2%, 0.4%, 0.6%, 0.8%, 1.0% and 1.5% of stevia stalk to study the effects of different stevia stalk concentrations on nutrient utilization and rumen fermentation in sheep (based on sheep diet). *In vitro* fermentation method was used, with 17 repetitions for each treatment. All fermentation substrate based on sheep diet with different stevia stalk concentrations were fermented for 2 h, 6 h, 12 h, 24 h and 48 h, then the gas production, dry matter degradability (DMD), crude protein degradability (CPD), neutral detergent fiber degradability (NDFD), acid detergent fiber degradability (ADFD), pH, ammonia nitrogen (NH_3_-N) and volatile fatty acids (VFAs) were determined.

**Results:**

The results showed that at different fermentation time, the change trend of gas production in each teatment was basically same, but the maximum occurred in 1.0% treatment at 48 h. The DMD, CPD, NDFD and ADFD of sheep diets increased with fermentation time increasing, especially the CPD_48h_, NDFD_48h_ and ADFD_48h_ of diets in 0.8%, 1.0% and 1.5% treatments were significantly higher than those in control (*P* < 0.05). The pH of fermentation substrate in each treatment remained within the normal range of 6.21∼7.25. NH_3_-N_24h–48h_in 0.8%, 1.0% and 1.5% treatments were higher than that in control. At 6 h–12 h, the total acid content of 0.8% and 1.0% treatments were significantly higher than those of other treatments (*P* < 0.05), it reached the highest in 1.0% treatment. According to overall evaluation, effect ranking of stevia stalk on sheep nutrient utilization was as follows: 1.0% >0.8% >1.5% >0.4% >0.6% >0.2%. Overall, 1.0% stevia stalk could promote nutrient degradation and sheep rumen fermentation.

## Introduction

Stevia (*Stevia rebaudiana* Bert.) is a green plant used as a calorie-free sweetener due to the presence of stevioside in leaves (Mehravaran et al., 2021), which has become the third natural sugar source widely used in food, beverage, medical, daily chemical and other industries in the world (Zhu, 2021; Sharma Saurabh et al., 2016), in which stevia leaves was only used as raw materials. Thus, a large number of stevia waste residues and discarded stevia stalks were produced as by-products of the sugar industry. Among them, only a small part of residues were used as agricultural fertilizers (Ding et al., 2016), others were used for fuel combustion or even landfill, which resulted in a lot of waste of straw resources and environmental pollution. Stevia was also rich in amino acids, minerals and active ingredients such as steviol and polyphenols (chlorogenic acid, flavonoids) (Xiong et al., 2022; Schiatti et al., 2022). It became a research hotspot in the field of livestock and poultry feed as feed ingredients. Studied found that adding 0.3%∼0.6% stevia (whole plant) into feed for one month could improve dairy cow appetite (Xie & Wang, 2010). 5% stevia residue regulated poultry digestive function (Shang, 2011). Stevia or stevia glycosides could improve appetite in livestock (Guo et al., 2016; Yu et al., 2020; Wang et al., 2011; Wang et al., 2014), goats (Han et al., 2019), poultry (Jiang et al., 2020) and racehorses (Ma & Ma, 2009), improve pets anorexia and feed conversion rate and regulate intestinal microflora balance (Ma & Ma, 2009). In addition, *in vitro* rumen fermentation of stevia residue was a typical acetic acid type fermentation, which could promote rumen carbohydrate fermentation, improve energy utilization and VFAs production, reduce methane production during rumen fermentation, all of which could be conducive to reducing greenhouse gas emission (He et al., 2017). Stevia could also stimulate the biological metabolic activity of rumen microbe, promote ammonia nitrogen transferring to microbial protein, so accelerate microbial protein synthesis (He et al., 2017). Therefore, stevia can be used as a natural green feed material with various bioactive functions. However, current studies on stevia have focused on dairy cows and poultry, and few have been reported in sheep. In this study, the effects of stevia stalk with different proportions on nutrients utilization in sheep rumen fluid and rumen fermentation were studied by *in vitro* gas production method. The *in vitro* gas production method is a research method to simulate the dynamic process of feed degradation in rumen in the laboratory and then determine the rate of nutrient degradation in feeds (Yang & Fang, 2015; Peñailillo et al., 2021). Therefore, we hypothesized that there would be differences in *in vitro* gas production and substrate degradation rates, etc., through adding different stevia stalk proportions to sheep rations, and determined the effect of stevia stalk on sheep nutrient utilization and rumen fermentation by *in vitro* gas production method, to determine the key indexes such as gas production rate and substrate degradation rate, so as to screen the best adding ratio and provide a theoretical reference for scientific application of stevia stalk in sheep production.

## Materials & Methods

### Test materials

Stevia stalk was collected from Wulan Town, Jingyuan County, Gansu Province, in November 2020. After drying at 65 °C for 48 h, initial moisture was determined, and plant material was crushed through a 40-mesh sieve for further use. Stevia samples were placed into nylon bags (9 cm × 5 cm, 400-mesh), which was placed into a 100 mL trachea with a chitin plug and plastic screw top. The basal diet was ground with a plant crusher and then prepared into dry matter substrate. The main nutrients in stevia stalk were shown in Table 1.

### Rumen fluid collection

All experimental protocols were approved by the Livestock Care Committee of Gansu Agricultural University (GAU-LC-2022-0555). Rumen fluid was obtained from animals at Zhonghua Sheep Farm in Lanzhou, and the experiment was conducted with the consent of the farmer. It was collected from three sheep (Small Tailed Han Sheep, 3 months old, male) at different sites in rumen, then mixed with CO_2_ gas in an insulated bottle preheated to 39 °C. After the rumen fluid was extracted, the experimental sheep were in good health and had no adverse reaction. The bottle mouth was immediately covered, then transported to the laboratory quickly. All of the mixed contents were filtered through four layers of gauze and stored in containers in a 39 °C water bath. CO_2_ gas was continuously injected, all of which were completed as soon as possible.

### Diet composition and nutrition

The trial sheep diet was made by Lanzhou Zhengda Co., Ltd. and formulated according to nutritional requirements of rams (body weight 45 kg, daily gain 50 g, fine to coarse ratio 4: 6) established by Agricultural Industry Standard of People’s Republic of China (NY/T816-2004). Dietary composition and nutritional information was shown in Table 2.

### Test design

A single-factor experiment was conducted in seven treatments, these were, 0%, 0.2%, 0.4%, 0.6%, 0.8%, 1.0%, and 1.5% (dry matter basis) proportions of stevia stalk, added to total mixed basal diet and mixed evenly for fermenting substrate. The specific test design was shown in Table 3.

**Table 1:** Stevia stalk powder nutrients (air-dried basis %).

**Material**	**DM(%)**	**CP**	**NDF**	**ADF**	**ESC**	**Ca**	**P**	**Ash**
**Stevia stalk**	96.64	3.76	57.62	40.04	10.90	0.73	0.13	5.34

**Notes.**

DM dry matter CP crude protein NDF neutral detergent fiber ADF acid detergent fiber ESC monosaccharide Ca calcium P phosphorus Ash ash

**Table 2:** Test diet composition and nutritional levels (dry basis).

**Formula composition**	**Proportion/ %**	**Nutritional level**	**Content**
Corn	38	DM / %	86.0
Corn germ meal	20	DE (MJ/kg)	14.23
Corn cob flour	9	ME (MJ/kg)	11.67
Rice Husk Powder	8	Ca (%)	4.3
Sprayed corn husk	6	P (%)	1.9
Corn husk	5	CP (%)	9.4
Cotton meal	3		
Rapeseed meal	2		
Soybean meal	3.5		
Bean curd	3.5		
1% Premix additives	1		
Salt	1		
Total	100		

**Notes.**

Each kilogram of premix contains: vitamin A, 220000 IU, vitamin D, 372000 IU, vitamin E, 2000 IU, D-biotin ,40.0 mg, nicotinamide, 2000 mg, Mn, 710 mg, Zn, 2005 mg, Fe, 830.0 mg, Cu, 680.0 mg, Co, 12 mg.

DM dry matter DE digestible energy ME metabolizable energy Ca calcium P phosphorus CP crude protein

**Table 3:** Test design.

Item	Control treatment	Test treatments					
Stevia stalk powder	0%	0.2%	0.4%	0.6%	0.8%	1.0%	1.5%
Stevia stalk powder (% of DM in diet) concentration (g/kg)	0	2	4	6	8	10	15

### *In vitro* fermentation method

Stevia stalk with concentrations of 0, 2, 4, 6, 8,10 and 15 g/kg was accurately weighed and placed in a homemade nylon bag (9 cm ×5 cm, 400-mesh) with 0.5000 g basal diet for substrate, numbered, sealed and then placed with forcep in the bottom of 100 mL tracheas, which were preheated to 39 °C for 30 min, in order to prevent gas from escaping, an appropriate amount of petroleum jelly was applied to the plunger of the syringe and then the filtered rumen fluid and artificial culture medium were mixed evenly at a volume ratio of 1: 2. 30 mL microbial culture mixture saturated with CO_2_ was accurately measured and placed in each trachea, sealed with rubber tube and clips, and the initial volume (mL) of each trachea was recorded. Each treatment contained 17 replicates. In order to ensure the representativeness of the test sample, three blank samples were made when the sample was incubated to eliminate test errors. After reading the initial volume, the tracheas were immediately transferred to water bath for culture preheated to 39  °C (the water surface height of the bath should be higher than liquid surface height of trachea culture solution) for 48 h. All data of the gas production at 2 h, 6 h, 12 h, 24 h, 36 h and 48 h were recorded. After 2 h, 6 h, 12 h, 24 h and 48 h of fermentation, the fermentation tube was placed into an ice water bath to stop fermentation. Three replicates were removed after 2 h, 6 h, 12 h and 24 h fermenting respectively, keeping the last five replicates for 48 h fermenting were placed into an ice water bath to stop fermentation, collected fermentation broth and nylon bags for index measurements.

### Indexes and measurement methods

#### Gas production

Gas production were recorded and the fermenting fluid were collected at 2 h, 6 h, 12 h, 24 h, 36 h, and 48 h; calculated cumulative gas production. The gas production for each time were calculated as fllows:

Gas production (mL/g) = (gas production in each tube recorded at each time - gas production in blank tubes at the same time)/fermentation substrate weight (Yang et al., 2017).

### Fermentation indexes

pH value of rumen fluid was determined by pH meter (P611 type). Determination of ammonia nitrogen concentration (NH_3_-N), 10 mL of rumen fluid was centrifuged at 3,500 r/min for 10 min, 2 mL of supernatant was taken and placed in 15 mL test tube, then 8 mL of 0.2 mol/L hydrochloric acid was added to 10 mL, shaking, and the content of NH_3_-N was determined by colorimetry (Feng & Gao, 2010).

For volatile fatty acids (VFAs), sample of each treatment at each time was centrifuged at 5,400 r/min for 10 min at 4 °C; 1 mL of supernatant was added to a 1.5 mL centrifuge tube containing 0.2 mL of 25% phosphonic acid solution containing internal standard 2-ethyl butyric acid. The samples were mixed well, placed in an ice water bath for more than 30 min, centrifuged at 10,000 r/min for 10 min, filtered through a 0.22 µL filter membrane, and detected by gas chromatography (Li, 2021).

### Rumen degradation rates

After 2 h, 6 h, 12 h, 24 h and 48 h of fermentation, nylon bag was washed with distilled water until clear. After natural drying, the nylon bag was transferred to an oven at 65  °C for 48 h and dried to a constant weight. The weight of the residue was weighed and used to determine DMD, CPD, ADFD and NDFD of different treatments at different times. ADF and NDF contents were determined by fiber washing method.

Every index of rumen degradation rate of feed was calculated as: (Mass of certain components in feed-the mass of associated components in residue)/the mass of associated components in feed ×100% (Liu et al., 2021).

### Data processing and statistical methods

Microsoft Excel 2010 was used for preliminary data collation. SPSS 23.0 software was used for single factor analysis of variance, duncan method was used for multiple comparisons. The results were expressed as means ± standard deviations, and *P* < 0.05 was considered significant. The data standardization of 48 h fermentation was transformed by membership function method.

The positive indexes were transformed according to the formula “Uij = (Xij-Ximin)/(Ximax-Ximin)”; the negative indexes were transformed according to the formula “Uij = 1 - (Xij-Ximin)/(Ximax-Ximin)”. In the formula Uij was the index (j) membership function value of different stevia stalk treatments (i); Xij was the measured value, Ximax and Ximin were the maximum and minimum measured values, respectively. The membership value of each index was obtained by the membership function method, and the average membership value of the measured index was calculated and used as the comprehensive evaluation of different stevia stalk treatments (Liao et al., 2022).

## Results

### Effects of stevia stalk on gas production, DMD, CPD, NDFD and ADFD of sheep diet

#### Effects of different stevia stalk proportions on gas production

**Figure 1:**
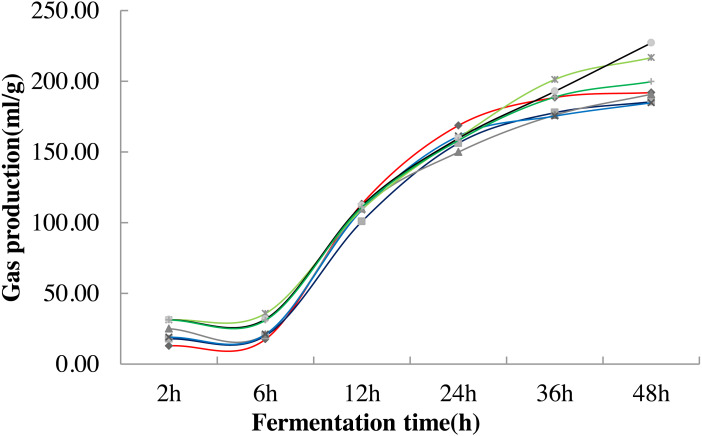
Variation curve of *in vitro* fermentation gas production under different stevia stalk treatments. Different lines in the graph indicate different treatments, red represents 0% treatment, dark blue represents 0.2% treatment, dark gray represents 0.4% treatment, blue represents 0.6% treatment, light green represents 0.8% treatment, black represents 1.0% treatment, and dark green represents 1.5% treatment.

The change trend of fermented gas production remained the same under different stevia stalk treatments. It tended to be slight at 2 h-6 h and increased at 6 h-24 h in each treatment, but they were significantly higher in 0.8%, 1.0% and 1.5% treatment at 2 h-6 h than that in the control (*P* < 0.05). All of treatment reached to flat at 36 h-48 h among treatments. The maximum value was obtained in 0.8% treatment at 36 h (*P* < 0.05) while got the highest value in 1.0% treatment at 48 h (*P* <  0.05) (Fig. 1; Table 4).

**Table 4:** Significance among treatments.

Item	Fermentation time					
	2 h	6 h	12 h	24 h	36 h	48 h
0%	c	d	a	a	ab	c
0.2%	c	c	b	ab	b	c
0.4%	ab	c	ab	b	b	c
0.6%	bc	c	ab	ab	b	c
0.8%	a	a	ab	ab	a	ab
1.0%	a	b	a	ab	ab	a
1.5%	a	b	a	ab	ab	bc
*P*- value	0.000	0.000	0.148	0.218	0.072	0.004

**Notes.**

Different lowercase letters in the same column indicate significant differences (*P* < 0.05) between different stevia stalk concentrations at the same time.

### Effects of stevia stalk on DMD, CPD, NDFD and ADFD

The DMD of sheep diet increased with fermentation time extention. It was lower in each treatment fermented at 2 h, 6 h and 12 h than that in the control. After 48 h of fermentation, there were no significant differences among treatments (*P* > 0.536). But the DMD_48h_ in 0.2% and 1.0% treatments were higher than that in the control (*P* > 0.05). CPD_12 h,_ CPD_24h_ and CPD_48h_ in 1.0% and 1.5% treatments were higher than that in the control (*P* < 0.05). And the ADFD_48h_ and NDFD_48h_ of 0.8%, 1.0% and 1.5% treatments were significantly higher than those of other treatments (*P* < 0.05) (Table 5).

**Table 5:** Effects of stevia stalk on DMD, CPD, NDFD and ADFD.

Items		Fermentation time (h)				
		2 h	6 h	12 h	24 h	48 h
DMD	0%	28.18 ± 1.14a	28.08 ± 0.06a	31.26 ± 0.85a	32.92 ± 0.02a	34.77 ± 0.78a
	0.2%	24.06 ± 0.36bc	24.45 ± 0.22bc	27.66 ± 0.25c	29.83 ± 0.04b	35.44 ± 0.27a
	0.4%	25.09 ± 0.92bc	24.91 ± 0.4c	28.34 ± 0.49bc	31.19 ± 0.19ab	34.96 ± 0.47a
	0.6%	25.59 ± 0.66b	27.63 ± 0.26a	29.67 ± 0.37abc	32.26 ± 0.21a	34.56 ± 0.51a
	0.8%	24.49 ± 0.42bc	24.88 ± 0.21bc	28.42 ± 0.03bc	31.37 ± 0.33ab	34.31 ± 0.79a
	1.0%	23.07 ± 0.17c	25.19 ± 0.76bc	27.64 ± 0.57c	31.39 ± 1.13ab	35.41 ± 0.29a
	1.5%	23.33 ± 0.41c	25.91 ± 0.57b	29.97 ± 1.36ab	32.95 ± 0.77a	34.19 ± 0.32a
*P*- value		0.002	0.000	0.018	0.013	0.536
CPD	0%	0.42 ± 0.001f	0.38 ± 0.01b	0.44 ± 0.017bc	0.51 ± 0.002bc	0.62 ± 0.002b
	0.2%	0.48 ± 0.004b	0.46 ± 0.001a	0.47 ± 0.001ab	0.51 ± 0.003bc	0.69 ± 0.007ab
	0.4%	0.38 ± 0.001 g	0.4 ± 0.007b	0.41 ± 0.014c	0.52 ± 0.009bc	0.63 ± 0.002b
	0.6%	0.43 ± 0.002e	0.46 ± 0a	0.45 ± 0.012abc	0.5 ± 0.008c	0.62 ± 0.004b
	0.8%	0.44 ± 0.002d	0.39 ± 0.003b	0.49 ± 0.002ab	0.58 ± 0.006b	0.73 ± 0.015a
	1.0%	0.51 ± 0.002a	0.44 ± 0.023a	0.5 ± 0.016a	0.66 ± 0.011a	0.77 ± 0.053a
	1.5%	0.46 ± 0.001c	0.44 ± 0.016a	0.5 ± 0.028a	0.67 ± 0.061a	0.77 ± 0.045a
*P*- value		0.000	0.001	0.004	0.000	0.002
NDFD	0%	38.8 ± 0.96a	41.9 ± 0.81cd	63.23 ± 0.67c	63.18 ± 0.96bc	58.03 ± 0.57b
	0.2%	31.93 ± 0.85bc	38.5 ± 0.6cd	58.38 ± 1.44d	57.83 ± 0.82cd	52.44 ± 1.85c
	0.4%	39.3 ± 1.21a	36.28 ± 0.4d	69.51 ± 0.56b	60.94 ± 0.55cd	55.93 ± 0.65c
	0.6%	30.1 ± 0.07c	42.79 ± 1.09ab	71.07 ± 1.06ab	69.75 ± 0.24a	58.25 ± 0.95b
	0.8%	34.23 ± 0.29b	46.19 ± 0.44a	66.18 ± 0.97c	54.8 ± 2.79d	71.13 ± 2.11a
	1.0%	24.44 ± 1.36d	36.37 ± 2.11d	63.05 ± 1.82c	60.07 ± 0.94cd	73.18 ± 1.01a
	1.5%	31.31 ± 0.5c	46.34 ± 1.41a	73.12 ± 0.43a	68.6 ± 3.9ab	72.4 ± 0.94a
*P*- value		0.000	0.000	0.000	0.001	0.000
ADFD	0%	26.52 ± 1.05bc	36.95 ± 0.15d	57.7 ± 0.42d	56.61 ± 0.34e	48.37 ± 0.49d
	0.2%	28.62 ± 0.49b	36.37 ± 0.34d	58.17 ± 0.07d	70.11 ± 0.22ab	45.73 ± 0.52e
	0.4%	22.53 ± 0.83de	36.37 ± 0.2d	60.99 ± 0.19c	59.79 ± 0.93d	45.45 ± 0.36e
	0.6%	24.33 ± 0.69cd	36.18 ± 0.61d	66.07 ± 1.01b	57.35 ± 1.6de	59.19 ± 0.44b
	0.8%	33.44 ± 0.32a	42.95 ± 0.21b	68.2 ± 0.18a	66.72 ± 1.34c	67.34 ± 0.71a
	1.0%	21.22 ± 1.42e	38.9 ± 0.79c	65.35 ± 0.76b	72.28 ± 0.99a	52.76 ± 1.09c
	1.5%	28.46 ± 0.42b	48.66 ± 0.31a	67.17 ± 0.86ab	68.58 ± 0.65bc	66.48 ± 1.35a
*P*- value		0.000	0.000	0.000	0.000	0.000

**Notes.**

Different lowercase letters in same column indicate significant differences (*P* < 0.05) between different stevia stalk concentrations at the same time and the same index; this also applies to the table below.

DMD dry matter degradability CPD crude protein degradability NDFD neutral detergent fiber degradability ADFD acid detergent fiber degradability

### Effects of stevia stalk on *in vitro* fermentation characteristics

As shown in Table 6, the pH value of rumen fluid derived from sheep treated with different stevia concentrations fluctuated between 6.21 and 7.25. It increased first and then decreased in 0%, 0.2%, 0.4% and 0.6% treatments while decreased in 0.8%, 1.0% and 1.5% treatments during fermentation. In short, with the extension of fermentation, the pH of rumen fluid declined. At 48 h, the pH was significantly lower in the 0.8% and 1.0% treatments than in the control (*P* < 0.05).

**Table 6:** Changes of pH, NH_3_-N and VFAs in rumen fluid in different Stevia stalk treatments (mmol L^−1^).

Items		Fermentation time (h)				
		2 h	6 h	12 h	24 h	48 h
pH	0%	6.98 ± 0.01b	7.25 ± 0.01a	6.85 ± 0.01a	6.63 ± 0.09b	6.48 ± 0.14bc
	0.2%	6.93 ± 0.01b	7.24 ± 0.01a	6.88 ± 0.01a	6.83 ± 0.01a	6.55 ± 0.1ab
	0.4%	6.93 ± 0.02b	7.21 ± 0.02ab	6.87 ± 0.01a	6.84 ± 0.01a	6.73 ± 0.01a
	0.6%	7.11 ± 0.1a	7.19 ± 0.01b	6.88 ± 0.02a	6.8 ± 0.03a	6.68 ± 0.07ab
	0.8%	6.9 ± 0.01b	6.83 ± 0.01c	6.57 ± 0.01c	6.6 ± 0.02b	6.24 ± 0.02d
	1.0%	6.89 ± 0.01b	6.81 ± 0.02c	6.62 ± 0b	6.56 ± 0.02b	6.21 ± 0.04d
	1.5%	6.92 ± 0.01b	6.83 ± 0.01c	6.6 ± 0b	6.52 ± 0.01b	6.29 ± 0.02cd
*P*- value		0.014	0.000	0.000	0.000	0.000
NH_3_-N	0%	20.08 ± 0.28ab	16.21 ± 1.98c	12.17 ± 0.33e	26.04 ± 0.24c	37.69 ± 0.87ab
	0.2%	20.93 ± 1.56ab	19.37 ± 0.51bc	17.53 ± 0.66d	23.93 ± 0.34de	37.27 ± 3.75ab
	0.4%	16.7 ± 1.26b	17.3 ± 2.64c	14.2 ± 1.37e	22.1 ± 0.57e	32.92 ± 1.16c
	0.6%	17.97 ± 1.99ab	15.44 ± 2.42c	20.7 ± 0.08c	24.88 ± 0.85cd	34.54 ± 1.17ab
	0.8%	18.94 ± 0.71ab	27.17 ± 1.96a	31.41 ± 0.56b	44.15 ± 0.72ab	38.67 ± 0.43b
	1.0%	22.48 ± 0.55a	24.53 ± 3.48ab	35.97 ± 1.07a	45.92 ± 0.3a	43.39 ± 1.91a
	1.5%	20.05 ± 3.15ab	27.85 ± 1.57b	30.48 ± 0.48b	42.39 ± 0.85b	43.67 ± 1.49a
*P*- value		0.294	0.004	0.000	0.000	0.000
Acetic acid	0%	12.27 ± 0.06c	18.2 ± 0.1d	18.81 ± 0.04d	16.69 ± 1.36d	24.51 ± 1.79ab
	0.2%	12.02 ± 0.52c	13.48 ± 0.14e	17.86 ± 0.14d	22.12 ± 1.03bc	13.89 ± 3.52c
	0.4%	16.22 ± 0.1b	13.67 ± 0.34e	18.52 ± 0.02d	23.51 ± 1.04bc	24.36 ± 4.07ab
	0.6%	11.42 ± 0.51c	12.02 ± 0.3f	20.07 ± 0.04c	28.52 ± 0.9a	16.88 ± 0.99bc
	0.8%	15.71 ± 0.03b	23.46 ± 0.56b	29.8 ± 0.64a	20.77 ± 0.43c	27.49 ± 0.74a
	1.0%	18.1 ± 0.17a	24.62 ± 0.31a	26.27 ± 0.43b	24.44 ± 0.57b	31.61 ± 1.17a
	1.5%	15.41 ± 0.14b	19.74 ± 0.25c	20.3 ± 0.56c	21.83 ± 0.41bc	22.27 ± 2.08abc
*P*- value		0.000	0.000	0.000	0.000	0.006
Propionic acid	0%	6.15 ± 0.13d	5.22 ± 0.01b	7.28 ± 0.21cd	5.17 ± 0.02d	15.74 ± 4.61bc
	0.2%	5.19 ± 0.11d	6.78 ± 0.23b	8.61 ± 0.11c	5.22 ± 0.01d	8.77 ± 1.74d
	0.4%	13.77 ± 0.25a	5.18 ± 0.01b	9.38 ± 0.03c	5.24 ± 0.01d	10.44 ± 0.88cd
	0.6%	5.56 ± 0.41d	5.19 ± 0.01b	5.13 ± 0.01d	11.62 ± 1.06c	8.69 ± 1.01d
	0.8%	8.46 ± 0.48c	19.46 ± 2.36a	18.64 ± 1.8b	17.92 ± 0.16b	20.04 ± 0.07ab
	1.0%	11.78 ± 0.76b	5.54 ± 0.21b	24.2 ± 0.45a	20.42 ± 0.67a	22.45 ± 0.63a
	1.5%	9.34 ± 0.66c	5.31 ± 0.1b	16.66 ± 1.23b	17.99 ± 0.3b	18.96 ± 1.55ab
*P*- value		0.000	0.000	0.000	0.000	0.000
Isobutyric acid	0%	0.48 ± 0.01bc	0.41 ± 0d	0.54 ± 0.01abc	0.69 ± 0.02ab	0.78 ± 0.13abc
	0.2%	0.48 ± 0.01bc	0.5 ± 0.01c	0.52 ± 0.01cd	0.47 ± 0.04e	0.56 ± 0.03c
	0.4%	0.5 ± 0ab	0.48 ± 0c	0.55 ± 0ab	0.57 ± 0d	0.6 ± 0.06bc
	0.6%	0.48 ± 0c	0.49 ± 0c	0.48 ± 0.04d	0.61 ± 0.02cd	0.63 ± 0.01abc
	0.8%	0.5 ± 0abc	0.59 ± 0.02a	0.61 ± 0.01b	0.72 ± 0a	0.84 ± 0ab
	1.0%	0.52 ± 0.02a	0.56 ± 0.01ab	0.68 ± 0.04a	0.72 ± 0.01a	0.86 ± 0.02a
	1.5%	0.5 ± 0.01abc	0.55 ± 0.01b	0.54 ± 0abc	0.65 ± 0bc	0.84 ± 0.03ab
*P*- value		0.011	0.000	0.000	0.000	0.033
Butyric acid	0%	1.94 ± 0.01b	2.19 ± 0.02c	3.02 ± 0.07de	3.48 ± 0.05e	4.67 ± 0.07ab
	0.2%	2.43 ± 0.23b	2.05 ± 0.03c	2.84 ± 0.05e	3.96 ± 0.04d	2.66 ± 0.62c
	0.4%	3.24 ± 0.05a	2 ± 0.01c	3.12 ± 0.06d	4.26 ± 0.02cd	4.37 ± 0.23ab
	0.6%	2.13 ± 0.32b	2.03 ± 0.02c	3 ± 0.1de	4.7 ± 0.23a	3.98 ± 0.22b
	0.8%	3.07 ± 0.04a	4.03 ± 0.32a	4.59 ± 0.04b	4.35 ± 0bc	5.06 ± 0.11ab
	1.0%	3.4 ± 0.24a	3.71 ± 0.07ab	5.44 ± 0a	4.59 ± 0.07ab	5.32 ± 0.05a
	1.5%	3.11 ± 0.06a	3.47 ± 0.14b	3.44 ± 0.05c	4.19 ± 0.09cd	4.86 ± 0.27ab
*P*- value		0.000	0.000	0.000	0.000	0.000
Isovaleric acid	0%	0.53 ± 0.01ab	0.52 ± 0b	0.56 ± 0.01cd	0.88 ± 0.14a	1.05 ± 0.16b
	0.2%	0.55 ± 0.02a	0.48 ± 0.02b	0.53 ± 0.01d	0.63 ± 0b	0.62 ± 0.12c
	0.4%	0.54 ± 0ab	0.49 ± 0.01b	0.57 ± 0.01cd	0.56 ± 0.04b	0.91 ± 0.05bc
	0.6%	0.49 ± 0.01bc	0.5 ± 0b	0.53 ± 0.01d	0.69 ± 0.03b	0.87 ± 0.09bc
	0.8%	0.52 ± 0ab	0.62 ± 0.02a	0.76 ± 0.02b	1.03 ± 0.03a	1.83 ± 0.14a
	1.0%	0.53 ± 0.01ab	0.59 ± 0.01a	0.83 ± 0.02a	1.03 ± 0a	1.6 ± 0.09a
	1.5%	0.45 ± 0.04c	0.58 ± 0.01a	0.58 ± 0.01c	0.9 ± 0.03a	1.46 ± 0.03a
*P*- value		0.023	0.000	0.000	0.000	0.000
Valeric acid	0%	0.56 ± 0b	0.66 ± 0.01c	1.02 ± 0.02d	1.74 ± 0.29a	2.51 ± 0.33a
	0.2%	0.71 ± 0.12b	0.63 ± 0.01c	0.94 ± 0.01d	1.36 ± 0.02b	1.08 ± 0.35c
	0.4%	1.01 ± 0.01a	0.63 ± 0c	1.05 ± 0d	1.54 ± 0ab	1.62 ± 0.06bc
	0.6%	0.64 ± 0.12b	0.62 ± 0.01c	0.92 ± 0.01d	1.77 ± 0.03a	1.47 ± 0.17bc
	0.8%	0.97 ± 0.01a	1.33 ± 0.09a	1.53 ± 0.02b	1.67 ± 0.01ab	2.09 ± 0.05ab
	1.0%	1.05 ± 0.06a	1.25 ± 0.01ab	1.72 ± 0.11a	1.77 ± 0.01a	2.14 ± 0.04ab
	1.5%	0.99 ± 0.01a	1.18 ± 0.03b	1.22 ± 0.01c	1.54 ± 0ab	1.91 ± 0.07ab
*P*- value		0.000	0.000	0.000	0.145	0.005
Total acid	0%	21.92 ± 0.04c	27.18 ± 0.12cd	31.23 ± 0.26de	28.64 ± 1.87d	49.05 ± 3.28ab
	0.2%	21.38 ± 0.8cd	23.92 ± 0.33de	31.3 ± 0.3de	33.75 ± 1.07c	33.36 ± 8.44c
	0.4%	35.28 ± 0.4a	22.45 ± 0.34e	33.19 ± 0.11d	35.69 ± 1.03c	42.31 ± 5.22bc
	0.6%	19.78 ± 0.58d	20.84 ± 0.29e	30.13 ± 0.1e	47.91 ± 1.87b	32.51 ± 3.2c
	0.8%	29.22 ± 0.42b	49.49 ± 3.36a	55.93 ± 1.2b	46.45 ± 0.37b	57.79 ± 0.65ab
	1.0%	35.38 ± 0.46a	36.27 ± 0.05b	59.14 ± 1.05a	52.96 ± 1.33a	63.62 ± 1.72a
	1.5%	29.79 ± 0.91b	30.83 ± 0.46c	42.73 ± 1.59c	47.1 ± 0.8b	50.3 ± 3.69ab
*P*- value		0.000	0.000	0.000	0.000	0.003

**Notes.**

NH_3_-N ammoniacal nitrogenin VFAs volatile fatty acids

The concentration of NH_3_-N in sheep rumen fluid decreased with the extension of fermentation time. 0.4% treatment had significantly lower NH_3_-N_48h_ than other treatments (*P* < 0.05), while there had no significant different NH_3_-N_48h_ between other treatments and control (*P* > 0.05).

During period of 2 h-48 h, the concentrations of acetic acid and propionic acid varied significantly among treatments, while the concentrations of isobutyric acid, butyric acid, isovalerate and valerate changed only slightly and remained relatively stable. At 6 h and 12 h, the total acid content of 0.8% and 1.0% treatments were significantly higher than that of other treatments (*P* < 0.05), while at 48 h, it had no difference in 0.8%, 1.0% , and 1.5% treatment, compared to the control. But showed the higest value in 1.0% treatment.

### Comprehensive analysis

It was insufficient to evaluate the fermentation characteristics with single index. Using the membership function method to analyze the relevant indicators of rumen fermentation characteristics comprehensively could reflect the overall performance of rumen fermentation accurately. In this study, the membership function method was used to comprehensively analyze pH, NH_3_-N, VFAs, DMD, CPD, NDFD and ADFD (Table 7). The higher the comprehensive analysis value, the better the fermentation of rumen nutrients. The results showed that order of the treatments was 1.0% >0.8% >1.5% >0.4% >0.6% >0.2%.

**Table 7:** Comprehensive indexes, weights, membership function values and rankings.

Item		0.2%	0.4%	0.6%	0.8%	1.0%	1.5%
Weights	pH	0.0542	0.0645	0.0615	0.0898	0.0909	0.0854
	NH_3_-N	0.0554	0.0767	0.0692	0.0843	0.0756	0.0715
	Acetic acid	0.0967	0.0674	0.0920	0.0771	0.0675	0.0912
	Propionic acid	0.0984	0.1010	0.1148	0.0679	0.0610	0.0688
	Isobutyric acid	0.0763	0.0871	0.0785	0.0803	0.0790	0.0769
	Butyric acid	0.0962	0.0716	0.0744	0.0798	0.0764	0.0796
	Isovaleric acid	0.0928	0.0773	0.0765	0.0496	0.0571	0.0596
	Valeric acid	0.1274	0.1038	0.1082	0.1039	0.1021	0.1083
	Total acid	0.0806	0.0776	0.0956	0.0740	0.0671	0.0808
	DMD	0.0538	0.0666	0.0638	0.0869	0.0861	0.0842
	CPD	0.0496	0.0659	0.0634	0.0737	0.0705	0.0670
	NDFD	0.0606	0.0695	0.0503	0.0706	0.0867	0.0664
	ADFD	0.0580	0.0713	0.0518	0.0621	0.0798	0.0603
Membership function value	pH	0.6538	1.0000	0.9038	0.0577	0.0000	0.1538
	NH_3_-N	0.4047	0.0000	0.1507	0.5349	0.9740	1.0000
	Acetic acid	0.0000	0.5909	0.1687	0.7675	1.0000	0.4729
	Propionic acid	0.0058	0.1272	0.0000	0.8249	1.0000	0.7464
	Isobutyric acid	0.0000	0.1333	0.2333	0.9333	1.0000	0.9333
	Butyric acid	0.0000	0.6429	0.4962	0.9023	1.0000	0.8271
	Isovaleric acid	0.0000	0.2397	0.2066	1.0000	0.8099	0.6942
	Valeric acid	0.0000	0.3776	0.2727	0.7063	0.7413	0.5874
	Total acid	0.0270	0.3148	0.0000	0.7974	1.0000	0.5714
	DMD	1.0000	0.6130	0.2965	0.3322	0.7904	0.0000
	CPD	0.4444	0.0689	0.0000	0.7334	0.9889	1.0000
	NDFD	0.0000	0.1681	1.0000	0.9013	0.2803	0.9622
	ADFD	0.0127	0.0000	0.6273	1.0000	0.3338	0.9604
Comprehensive evaluation value		0.1372	0.3139	0.2838	0.7041	0.7355	0.6559
Total ranking		6	4	5	2	1	3

## Discussion

### Substrate degradation rate

Dry matter degradation rate, gas production and *in vivo* digestibility are highly correlated (Blümmel, Makkar & Becker, 1997). In this experiment, the trend of gas production with different concentrations was consistent, and they were stable at 2 h-6 h, it was because the rumen microbial activity was weak and a small amount of gas produced in early fermentation stage, while to 6 h-36 h, rumen microbe proliferated, acting on substrate completely to be digested enough, thus producing large amounts of gas rapidly. The gas production decreased slowly with the nutrients being consumed. Menke et al. (1979) showed that the rate of forage organic matter degradation was positively correlated with gas production during *in vitro* incubation, *i.e.,* higher gas production indicated higher forage degradation in rumen and higher microbial activity. The results of this study showed that the CPD_48h_, NDFD_48h_ and ADFD_48h_ of 0.6%, 0.8%, 1.0% and 1.5% stevia stalk treatments were higher than those without stevia stalk, and 1.0% treatment has the highest gas production at 48 h, which was consistent with the result of DMD_48h_, CPD_48h_ and NDFD_48h_. It showed that appropriate concentration of stevia stalk could improve sheep rumen digestive function and promote crude fiber digestion and absorption in diet, adding 1.0% stevia stalk could promote the microbe fermentation activities in rumen more effectively, thus improved feed digestibility. Studies showed that the active ingredients such as stevia or flavonoids in *stevia rebaudiana* could reduce rumen protozoa and increase cellulolytic bacteria amount to facilitate fiber decomposition and change rumen micro ecological environment (He et al., 2017). This was consistent with the results of the Sarnataro’s study (2020). Studies reported that sea buckthorn flavonoids (Bai et al., 2020) and sea onion flavonoids (Bao et al., 2015) significantly increased rumen gas production, which was consistent with the results of the present experimental study. Its specific mechanism needs further research in feeding experiments.

### Rumen fermentation characteristics

The pH value is a comprehensive index reflecting rumen fermentation level, it not only reflects the strength of rumen microbial activity but also serves as an important indicator to evaluate nutrients decomposition status and assess rumen internal environment stability. The normal rumen pH is 5.5 ∼7.5, and pH values higher or lower than this range will affect normal fermentation in rumen. When pH value is lower than 6.0, the number of fiber-decomposing bacteria and protozoa will decrease, and the cellulose and protein degradation rates in diet will also decrease (Liu et al., 2012; Krause & Oetzel, 2005). In this experiment, the pH range was 6.21 to 7.25, and high stevia stalk concentration (0.8%, 1.0%, 1.5%) could decrease the pH of the rumen culture medium, but the final pH still remained within the normal physiological range. This was consistent with the results of He’s study (2017). It indicated that the artificial rumen was in a normal fermentation state and had not adverse effects on normal metabolism of rumen microbe, ensuring rumen microbe normal growth and reproduction. An explanation was that rumen microbe decomposed starch and structural carbohydrates in feed, producing a large number of VFAs. Moreover, with the extension of fermentation, VFAs was accumulated in fermentation tubes, resulting in a further decline in pH value. Consistent with the result of a negative correlation between VFAs and rumen pH by Stritzler et al. (1998).

The NH_3_-N concentration in rumen is an important indicator of nitrogen metabolism, microbial protein synthesis and protein degradation. Maintaining an appropriate NH_3_-N concentration is a prerequisite for microbial protein synthesis in rumen (Feng et al., 2017). A suitable rumen NH_3_-N concentration is beneficial to growth of microbe, and higher NH_3_-N concentration is beneficial to fibrolytic bacteria growth and reproduction and DM degradation. The most suitable NH_3_-N concentration range for microbial activity was 0.35 ∼29 mg/dL (Galdi et al., 2002). In this experiment, the NH_3_-N concentration was 12.17 ∼45.92 mg/dL, and NH_3_-N concentrations were relatively high in all treatments for 48 h. He et al. (2017) found that stevia residue reduced ruminal NH_3_-N concentration, contrary to the results of the present study. This might be related to factors such as feed type, feeding form and *in vitro* fermentation. In this experiment, after 12 h of rumen fermentation, NH_3_-N concentration in each treatment increased significantly, which might be due to the longer retention time of chyme in rumen and the longer interaction time between rumen microbe and feed particles (He et al., 2020). This might also be associated with lower microbial utilization. With the extension of fermentation, the pH of rumen fluid decreased, and the decreased pH environment was not conducive to the growth of microbe that decomposed structural carbohydrates. The utilization efficiency of NH_3_-N by microbe that decompose structural carbohydrates decreased, resulting in increasing of NH_3_-N concentration in rumen. This was also consistent with the decreasing trends of the pH value.

VFAs in the rumen are important indicators of rumen fermentation in sheep and can reflect the environmental conditions in the rumen, the fermentation degree and the type of feed in rumen (Liu et al., 2012). In this experiment, at 48 h of fermentation, the content of acetic acid and propionic acid increased in all treatments, and acetic acid contents were higher than propionic acid contents, and the total acid content of 1.0% treatment was the highest. It could be seen that adding 1.0% stevia stalk could increase the concentration of VFA *in vitro* fermentation, better improve rumen fermentation capacity, maintain the normal growth, development and reproduction of rumen microbe and ensure the normal function of rumen, which was consistent with the results of pH value decreased to the lowest and NH_3_-N concentration increased. However, Jiang (2019) found that the addition of stevia pellets to lactating cows did not affect rumen fermentation. Studies had shown that *in vitro* rumen fermentation of stevia residue was a typical acetic acid type fermentation, which could promote rumen carbohydrate fermentation, improve energy utilization and VFAs production (He et al., 2017). This indicated that stevia stalk also had good feeding value as a by-product. Stevia extract could reduce the number of rumen protozoa, thereby affecting rumen metabolism (Chiara & Mauro, 2020). It was also reported that stevia could help digest and prevent constipation. Stevia could promote the proliferation of bifidobacteria and lactobacillus in human body, improve human immunity, prevent and treat constipation and diarrhea (Xu, 2013). *Stevia rebaudiana* residue could significantly inhibit the growth of harmful bacteria and promote the proliferation of beneficial bacteria (Zhong et al., 2022). Therefore, it was speculated that the glycosides in stevia stalk also had an effect on sheep gastrointestinal fermentation. However, whether the increase of VFAs content in this experiment was related to steviosides in *stevia rebaudiana*, its mechanism needs further study.

## Conclusions

Evaluating the nutritional value of ruminant feed by *in vitro* gas production was feasible, and adding stevia to the diet could be used by ruminants and could improve feed conversion, promote rumen fermentation and, to a certain extent, reduce breeding costs. Under the experimental conditions, the appropriate level of stevia stalk supplementation was 1.0%.

##  Supplemental Information

10.7717/peerj.14689/supp-1 Supplemental Information 1 Raw data used for all indicators in this article

10.7717/peerj.14689/supp-2 Supplemental Information 2 The ARRIVE guidelines 2.0: author checklist

## Additional Information and Declarations

### Data Availability

The following information was supplied regarding data availability: The raw measurements are available in the Supplementary File.
